# Fecal miRNome and Proteome Profiling Uncovers Stage-Specific Biomarkers of Alzheimer’s Disease in 3×Tg-AD Mice

**DOI:** 10.1007/s10571-026-01735-5

**Published:** 2026-05-11

**Authors:** Roberta Vitali, Barbara Tanno, Arianna Casciati, Francesca Palone, Luisa Pieroni, Marta Morotti, Massimo Santoro, Emiliano Fratini, Simonetta Pazzaglia, Maria Vittoria Podda, Mariateresa Mancuso

**Affiliations:** 1 Biotechnologies Division, National Agency for New Technologies, Energy and Sustainable Economic Development (ENEA), 00123 Rome, Italy; 2 Departmental Faculty of Medicine, UniCamillus-Saint Camillus International University of Health and Medical Sciences, 00131 Rome, Italy; 3 IRCCS San Camillo Hospital, 30126 Venice, Italy; 4 Fondazione Policlinico Universitario A. Gemelli IRCCS, Rome, Italy; 5 Department of Neuroscience, Università Cattolica del Sacro Cuore, 00168 Rome, Italy

**Keywords:** Multiomics, Stool, Intestinal barrier dysfunction, Gut-brain axis, Early diagnosis, miR-146b-5p, IgKappa, Elastase3B

## Abstract

Reliable, minimally invasive biomarkers for Alzheimer’s disease (AD) remain a critical unmet need. Considering mounting evidence for gut–brain interactions in AD, we explored whether fecal samples contain host-derived molecular signals that reflect disease-related changes and could serve as accessible biomarkers. Fecal small RNA sequencing and quantitative proteomics were performed in symptomatic 15-month-old 3×Tg-AD mice and controls. Pathway and network analyses identified key dysregulated molecules. Selected miRNAs and proteins were validated by qPCR or Western blotting in independent pre-symptomatic, early symptomatic, and severe cohorts. To assess whether fecal signals mirrored tissue alterations, colon samples were analyzed for tight-junction proteins, inflammatory markers, epigenetic regulation, and expression of selected miRNAs and proteins. Multi-omics identified 31 miRNAs and 81 proteins modulated in AD fecal sample, revealing a distinct AD-associated fecal signature involving pathways linked to neuroinflammation, synaptic dysfunction, vascular imbalance, and metabolism. Several miRNAs— including miR-322-5p, miR-194-1 and miR-223-3p were significantly altered and validated across disease stages (3-,7-,15-old months mice, respectively). Notably, miR-146b-5p expression is inversely correlated with AD severity, exhibiting a stepwise decline from asymptomatic individuals to those with mild and severe stages. IgKappa and Ela3B emerged as the strongest protein candidates, showing opposite and progressive modulation. Notably, IgKappa and miR-146b showed the most consistent trajectories. Colon analyses uncovered reduced Claudin-7, strong IgKappa upregulation driven by enhancer hypomethylation, and partial overlap between fecal and tissue miRNA profiles, indicating that fecal signals reflect subtle epithelial and immune perturbations associated with AD. Multi-omics at severe stage and a stage specific validation support fecal miRNAs and proteins as promising, host-derived biomarkers for AD. Fecal profiling offers a practical, repeatable, and low-cost approach with translational potential for early detection and longitudinal monitoring in neurodegeneration.

**Graphical Abstract:**

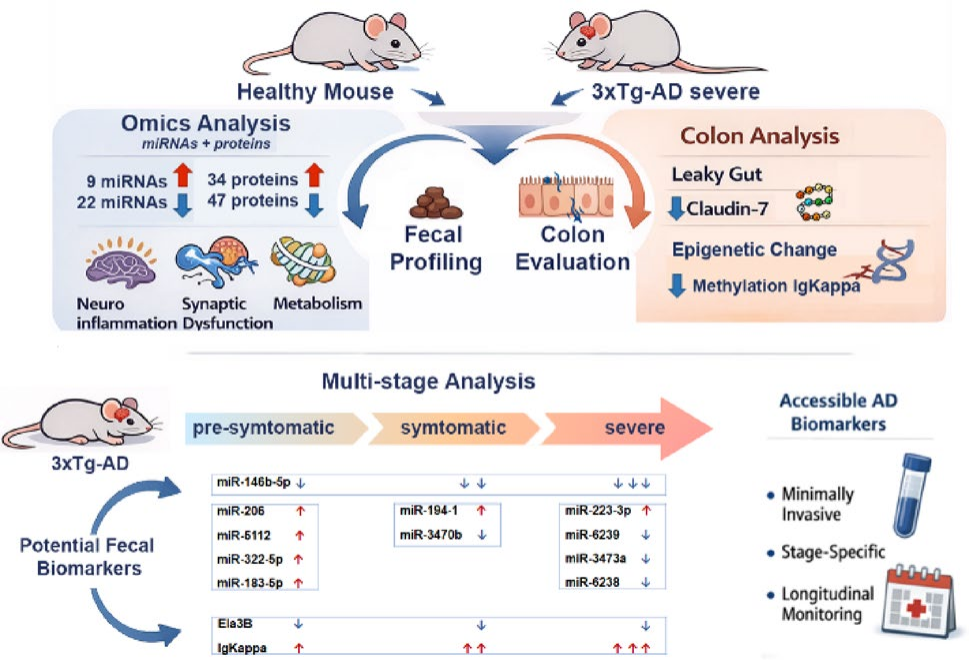

**Supplementary Information:**

The online version contains supplementary material available at 10.1007/s10571-026-01735-5.

## Background

Alzheimer’s disease (AD), the most common cause of dementia worldwide (World Health Organization 2021), is a progressive neurodegenerative disorder defined by cognitive decline accompanied by amyloid-β (Aβ) accumulation and tau pathology (Grimm and Hartmann 2012), (Breijyeh and Karaman 2020). Contemporary models conceptualize AD as a pathological continuum in which a prolonged preclinical phase precedes the onset of mild cognitive impairment and, eventually, overt dementia. During this clinically silent period—which may extend 6–10 years—subtle molecular, cellular, and physiological abnormalities progressively emerge (Insel et al. 2016; Vermunt et al. 2019). Although neuroimaging and cerebrospinal fluid (CSF) biomarkers, including MRI, [18 F] FDG-PET, amyloid- and tau-PET, and CSF Aβ42, p-tau, and t-tau, have substantially improved diagnostic capability (Jack et al. 2018; Leuzy et al. 2021), these methods remain invasive, costly, and generally impractical for population-level or repeated early-stage screening.

In recent years, accumulating evidence implicates the gut–brain axis in AD pathogenesis. Gut microbiota dysbiosis has been shown to modulate systemic and central nervous system inflammation, promoting microglial activation and contributing to amyloid-β aggregation and tau hyperphosphorylation. Microbial-derived metabolites (e.g., short-chain fatty acids) and endotoxins can influence blood–brain barrier integrity and neuroimmune signaling through bidirectional gut–brain communication. These findings support a mechanistic link between intestinal homeostasis and neurodegenerative processes in AD (Bou Zerdan et al. 2022; Liang et al. 2023a, 2023b; Xie et al. 2023; Yang et al. 2024).

Fecal samples provide a non-invasive window into this bidirectional communication, capturing microbial composition as well as metabolites, host-derived proteins, and microRNAs (miRNAs) that reflect alteration in intestinal immunity, epithelial barrier integrity, and neuroimmune signaling. While alterations in the gut microbiome and metabolome have been documented across AD models and patient cohorts (Afrin et al. 2025), the regulatory molecular layer in stool—particularly fecal miRNAs and proteins—remain largely uncharacterized. This gap limits our understanding of gut-derived molecular pathways linked to AD and hinders the development of accessible biomarkers suitable for early detection and disease monitoring.

Building on this rationale, we hypothesized that AD-associated gut dysregulation would be reflected in the fecal molecular milieu, and that specific miRNAs and proteins in stool could serve as non-invasive biomarkers sensitive to disease stage. To test this, we utilized the triple-transgenic 3×Tg-AD mouse model, which develops both Aβ and tau pathology (Oddo et al. 2003) and recapitulates key phases of disease progression: early molecular changes without cognitive impairment at approximately 3 months, emerging cognitive deficits with the onset of intraneuronal Aβ accumulation in the hippocampus at 6–7 months, and progressive synaptic and cognitive decline with aging (Cocco et al. 2020; Sterniczuk et al. 2010). We first performed comprehensive profiling of fecal miRNAs and proteins in 15-month-old mice exhibiting overt pathology, then selected and validated candidate biomarkers in independent cohorts spanning 3, 7, 15, and 24 months to map their temporal trajectories. To determine whether fecal signatures aligned with intestinal pathology, we additionally carried out histological, molecular, and epigenetic analyses of colon tissue in aged 3×Tg-AD and control mice.

Together, these experimental approaches identify stool-derived miRNAs and proteins that change systematically with AD progression and support their promise as non-invasive biomarkers for monitoring disease onset, prognosis, and gut involvement in AD.

## Materials and Methods

### Ethics Approval

All experiments and animal procedures were approved by the Catholic University Ethics Committee and were in line with the Italian (Ministry of Health guidelines, Legislative Decree No. 116/1992) and European Union (Directive No. 86/609/EEC) legislation on animal procedures (animal protocol #17-23, approved by the Ministry of Health, number 264/2019-PR). All efforts were made to limit the number of animals used and to minimise suffering.

### Murine Model of 3×Tg-AD and Sample Collection

Female wild-type (WT) (B6129SF2/J) and 3×Tg-AD mice (aged 3, 7, 15 and 24 months) were used in this study. 3×Tg-AD mice harbor three mutations associated with familial AD (APP K670_M671delinsNL (Swedish), Tau (MAPT) P301L and Presenilin-1 (PSEN1 M146V)). The colonies were established in-house at the Animal Facility of the Università Cattolica from breeding pairs purchased from the Jackson Laboratory. Mice were housed under standard animal housing conditions with a 12-hour light/dark cycle, constant humidity (60–75%), controlled ambient temperature (20–22 °C) and free access to food and water. For stool collection, individual mice were placed in empty clean cage without bedding. Mice were allowed to defecate naturally and the first two-three fecal pellets per animal were collected using a sterile toothpick and placed in a 1.5 ml Eppendorf. Fecal pellets were then stored at −80 °C until further analysis.

Colon samples were obtained from 24-month-old mice and age-matched controls. Mice were deeply anesthetized by intraperitoneal injection of ketamine (87.5 mg/kg) and xylazine (12.5 mg/kg) and transcardially perfused with ice-cold PBS. Colon tissues were then processed for immunoblotting, histological analyses or DNA and RNA extraction.

### Experimental Design

Fecal samples from 15-month-old mice (*n* = 7 AD; *n* = 6 controls) were initially analyzed by omics approaches to identify dysregulated miRNAs and proteins. Data acquisition and statistical analyses were performed with investigators blinded to the experimental groups. Candidate biomarkers were then validated in an independent cohort representing key stages of disease progression, including pre-symptomatic (3-month-old, *n* = 4 AD; *n* = 4 controls), early symptomatic (7-month-old, *n* = 8 AD; *n* = 6 controls), and severe disease stages (24-month-old, *n* = 10 AD; *n* = 7 controls). Fecal samples from the 15-month-old cohort used in the discovery omics analysis were also included in the validation phase.

**Fig. 1:**
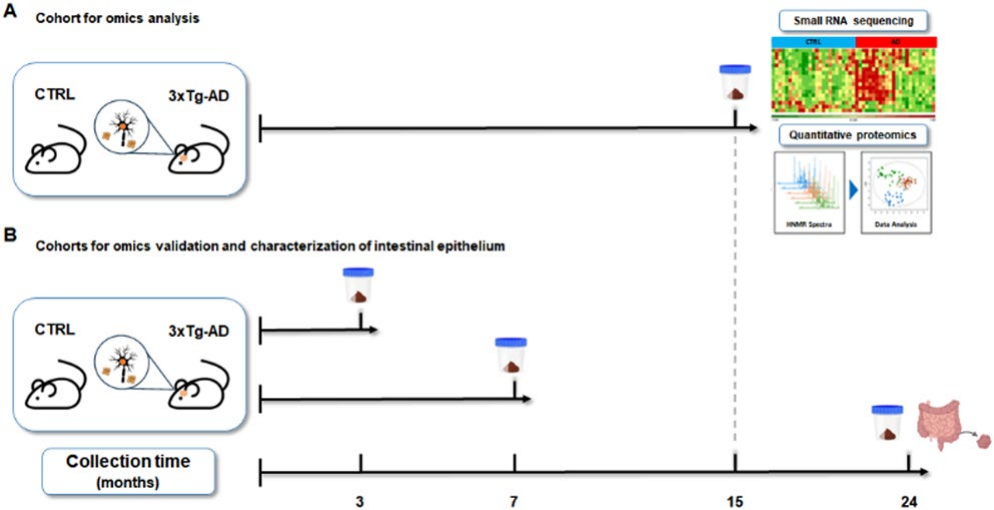
Experimental design. This figure provides an overview of the experimental groups and sampling strategy employed in the study. **A** Fecal sample collection from control (*n* = 6, CTRL) and 3×Tg-AD mice (*n* = 7) at 15 months of age for comprehensive multi-omics analysis. **B** Experimental groups used for fecal and colon tissue collection performed to validate the multi-omics findings and to conduct detailed morphological and molecular characterization of the intestinal epithelium

Colon tissue samples were collected from 24-month-old mice (*n* = 10 AD; *n* = 7 controls) for histological, molecular and epigenetic analyses. An overview of the experimental design is provided in Fig. 1.

### miRNome Analysis and PCR Array (Custom Plates)

Murine stool samples were weighed, and 30 mg of each sample was used to extract total RNA extraction using miRNeasy kit (QIAGEN, Milan, Italy) according to the manufacturer’s instructions. RNA quantification was performed using the Agilent TapeStation 200 (Agilent 5301 Stevens Creek Blvd. Santa Clara, CA 95051, USA).

miRNome NGS was performed by QIAGEN s.r.l. Heidelberg as follow described. RNA (100 ng) was converted into miRNA NGS libraries using QIAseq miRNA Library Kit (QIAGEN). Based on insert quality and concentration, libraries were pooled in equimolar ratios and sequenced using the NextSeq instrument (Illumina Inc. 5200 Illumina Way, San Diego, USA) according to the manufacturer’s instructions. On average, 77 million reads were obtained per sample.

Sequencing reads were aligned using the miRBase database and a small RNA reference database, to identify all miRNAs present in fecal samples. By comparing miRNA expression profiles, AD vs. CTR, we identified 31 differentially expressed miRNAs (DEGs). Only statistically significant miRNAs (*p* ≤ 0.05, log fold-change ≥ 0.7) were used for gene/miRNA enrichment analysis using Cytoscape plug-in “ClueGo” (v.2.1.7) and “CluePedia” (v.1.1.7) (29) with a validated miRTarBase score > 0.6.

Top 20 predicted target genes for each miRNA were selected to identify the affected pathways and functions using the REACTOME database (https://reactome.org). Pathways were considered when they contained at least three genes and ≥ 4% representation.

miRNAs with a fold change > 2 were selected (*n* = 25) to design a miRCURY LNA miRNA Custom Panel (QIAGEN), including 25 selected miRNAs, 5 housekeeping and 2 positive PCR controls. A total of 100 ng of RNA was reverse-transcribed using the miRCURY LNA RT Kit (QIAGEN) and reactions were performed in a real-time PCR cycler according to the manufacturer’s instructions. Results were expressed as fold change comparing AD *versus* CTRL.

### Fecal Protein Extraction and Quantification

Murine stool specimens, stored at −80 °C, were weighed and re-suspended in Extraction Buffer 1X (Eurospital SPA, Trieste, Italy) to obtain a final concentration of 100 mg/ml. Samples were vortexed and solubilized by keeping in a vigorous orbital shaking for 1 h at room temperature, followed by 2 centrifugation steps for 10 min at 10.000 rpm at 4 °C. Protein concentration was determined by Bradford assay (Bio-Rad Laboratories, Hercules, CA). Extracted were stored at −80 °C until proteomic analyses or validation by immunoblotting.

### Comparative Proteomics with LC-HDMS^E^ Shotgun Analysis

Proteins extracted from the fecal samples were analyzed using a bottom up shotgun proteomic approach. Briefly, 50 µg of each protein extract from each sample (6 control mice and 7 AD mice) was digested with trypsin using the Filter-Aided Sample Preparation (FASP) method (Marini et al. 2020). Lyophilized peptides were resuspended in 50 µl 0.1% formic acid (FA) and 350ng of each sample, spiked with 500fmol of Hi3 *E. Coli*ClpB (Waters) as internal standard, were used for LC-MS analysis.

Peptides were analysed using a Symmetry C18 Trap Column 100 Å, 5 μm, 180 μm × 20 mm (Waters) and were separated in Liquid Chromatography by a 125 min reverse phase gradient at 1.2 µl/min (linear gradient, 2–40% ACN over 90 min) using a HSS T3 100 Å 1.8 μm, 150 μm × 100 mm iKey (Waters) maintained at 40 °C on an ACQUITY M Class UPLC system (Waters). Separated peptides were then analysed in a bottom-up experiment on a Synapt G2-Si Mass spectrometer (Waters) directly coupled to the chromatographic system. Data were acquired in High Definition MS^E^ (HD MS^E^), a data independent acquisition (DIA) protocol where ion mobility separation (IMS) has been integrated into LC- MS^E^ workflow (Distler et al. 2016). Mass spectra were acquired in positive polarity and resolution analyser mode. TOF MS was operating over 50–2000 m/z using a scan time of 0.5 s and a continuum data format. Data were post-acquisition lock mass corrected using the doubly charged monoisotopic ion of (Glu1)-Fibrinopeptide B (Waters) sampled every 30 s. For IMS, wave height at 40 V, wave velocity of 1000 m/s and transfer wave velocity of 175 m/s were applied. Instrument settings were defined to apply a drift time specific transfer collision energy ramp as previously described (Vacca et al. 2023). Data from three replicate experiments for each sample were processed for qualitative and quantitative analysis using the Progenesis QI for Proteomics v4.1 software (Waters). The qualitative identification of proteins was obtained by searching in the UniProt database restricted to mouse taxonomy (UniProt 2022_04, restricted to Mus musculus (Mouse taxonomy), to which the sequence of the EColi ClpB protein (UniProt ID: P63285) was appended. Search parameters were set as: 6 ppm for peptide tolerance and 10 ppm for fragment tolerance, minimum one fragment ion matched per peptide, minimum three fragment ions matched per protein, minimum one peptide matched per protein, one missed cleavage accepted, carbamidomethylation of cysteines as fixed modification and oxidation of methionines as variable modification, false discovery rate (FDR) of the identification algorithm at 1%. Label-free quantitative analysis was obtained by using the “Absolute quantification using HiN” option integrated in the software (Silva et al. 2006) that averaged the most abundant n peptides (*n* = 3) for each protein to provide a reading for the protein signal, using the Hi3 E. coli standard (Waters) as reference. Differentially expressed proteins were selected with a threshold for significance set to p-values ≤ 0.05 (≥ 1.3 in -log 10 scale) and |log 2 (MFC)| ≥ 0.4 (equal to 1.5-fold increase/decrease). For graphical representation of expression analysis, we used open source webapp HEATMAPPER (Silva et al. 2006) and VolcanoseR (Goedhart and Luijsterburg 2020) to produce respectively Heat Map and Volcano Plots.The Kyoto Encyclopedia of Genes and Genomes (KEGG) enrichment analysis was performed to identify signaling pathways using Enrichr platform (Evangelista et al. 2023). The mass spectrometry proteomics data have been deposited to the ProteomeXchange Consortium (Deutsch et al. 2023) via the PRIDE (Perez-Riverol et al. 2025) partner repository with the dataset identifier PXD070630 and 10.6019/PXD070630.

### Integrated miRNome-Proteome Network Analysis

Differentially expressed miRNAs and proteins between the 3×Tg-AD and control group were identified through miRNome and proteome analyses, using a threshold of p-value ≤ 0.05 and fold change ≥ 1.5. Experimentally validated mouse miRNA–mRNA interactions were retrieved from miRTarBase V9.0 (Huang et al. 2022). A miRNA–mRNA interaction subnetwork was constructed in Cytoscape v3.10.1, by incorporating the differentially expressed miRNAs with their target mRNAs. A refined sub-subnetwork was subsequently generated by retaining only those mRNAs encoding protein identified as differentially expressed in the proteomic analysis.

### Immunoblot Analysis

For Western blot analysis, colon tissues were lysed in ice-cold lysis buffer containing 50 mM Tris (pH 7.4), 5 mM EDTA, 250 mM NaCl, 0.1% Triton X‐100, 1 mM phenylmethylsulfonyl fluoride, 5 mg/ml aprotinin, 5 mg/ml leupeptin, and 1 mM sodium orthovanadate. Total proteins extract from colon tissue or fecal samples were analyzed using antibodies against IgKappa chain (OX20; sc-53080, Santa Cruz Biotechnology, Inc., CA, USA; dilution 1:500), Ela3B (#MBS2003441, MyBioSource, CA, USA; dilution 1:1000), Claudin-7 (ab27487, RRID: AB_470990, Abcam, UK; dilution 1:1000), ZO1 (ZO1-1 A-12; 33-9100, RRID: AB_2533147, Invitrogen Waltham, MA USA; dilution 1:1000) and βActin (AC-15) (A3854, RRID: AB_262011, Sigma, St. Louis, MO; dilution1:5000). Antibody specificity has been validated by the manufacturers reported in previous studies. (Mason et al. 1981; Wu et al. 2017; Tang et al. 2024; Ma et al. 2013; Basile et al. 2023).

### Histological Analysis: Assessment of Goblet Cell Number and Immunohistochemistry

Distal colon tissues were dissected, fixed in 10% buffered formalin and embedded in paraffin wax according to standard procedures. For goblet cell analysis, tissue sections were stained with Periodic Acid Shift (PAS) reagent (Bio-Optica, Milan, Italy) following standard protocols. Goblet cells were quantified as the number of PAS-positive cells per crypt using NIS-Elements BR software (Nikon Instruments Europe B.V.). Two sections per animal were analyzed.

For immunohistochemistry, 4-µm paraffin-embedded colon sections were prepared according to standard procedure. After deparaffinization, antigen retrieval was performed by microwave heating in Citrate Buffer (pH 6.0) (Abcam ab93678, UK). Sections were washed in distilled water for 5 min and endogenous peroxidases activity was blocked by incubation in 3% H_2_O_2_ for 10 min. Sections were then incubated with 5% bovine serum albumin (Santa Cruz Biotechnology, Santa Cruz, CA, USA) for 30 min and incubated with anti-Iba1 primary antibody (ab178846, RRID: AB_2636859, Abcam) at a dilution of 1:2000, overnight at 4 °C in a humified chamber. After incubation with the biotinylated goat anti-rabbit secondary antibody (ab205718, RRID: AB_281960, Abcam; dilution 1:200) antibody-antigen complexes were visualized using the Vectastain^®^ Elite^®^ ABC Kit and Vector NovaRED substrate (VectorLaboratories, CA, USA). Sections were counterstained with hematoxilyn. Quantitative analysis of IBA1 immunoreactivity was performed using HistoQuest^®^ 6.0 software (TissueGnostic, Vienne, Austria). Two fields per section were analyzed for each animal, and IBA1 levels were expressed as the percentage of IBA1-positive area relative to the total area of the colon section (µm²). Antibody specificity has previously been validated by manufacturer and reported in the literature (Rangaraju et al. 2018; Morgan et al. 2018).

### DNA and RNA Isolation

Colon tissue fragments were collected from each mouse and immediately snap-frozen in liquid nitrogen. DNA and total RNA were extracted using the AllPrep DNA/RNA Mini Kit or the miRNeasy kit (QIAGEN) according to the manufacturer’s instructions. Nucleic acid concentration was determined using NanoDrop (Thermo Fisher Scientific).

### Real-Time PCR

Total RNA (1 µg) was reverse transcribed by IScriptTM cDNA Synthesis Kit (BioRad, Hercules, CA, USA). Quantitative PCR was performed using a CFX96 TouchTM Real-Time PCR Detection System (Bio-Rad) with SsoAdvanced Universal SYBR Green Supermix (BioRad). Primers for Claudin-7, IgKappa, Ela3B and GAPDH are listed in Supplementary Table 1. Relative gene expression levels were calculated using 2 − ΔΔCt method and GADPH used as the reference gene.

miRNA expression analysis was performed using TaqMan^®^ miRNA Assay (TermoFisher Scientifi) for hsa-miR-142-5p (#Assay ID:002248), mmu-miR-709 (#Assay ID:001644), hsa-miR-206 (#Assay ID:000510), hsa-miR-146b (#Assay ID:001097), mmu-miR-6236 (#Assay ID:474656_mat), mmu-miR-8117 (#Assay ID: 466365_mat), mmu-miR-194-1*(#Assay ID:462509_mat), hsa-miR-183 (#Assay ID: 002269) and for U6 snRNA (# Assay ID: 001973) as the endogenous control.

All reactions were performed in technical triplicate for each biological replicate and the relative expression levels were calculated using the 2 − ΔΔCt method. Quantitative method was used to normalize the reference gene and to calculate the relative expression of target genes.

### Epigenetic Analysis

DNA methylation analysis was performed following bisulfite conversion of genomic DNA. Briefly 1 µg of genomic DNA in 20 µl was treated using the EZ DNA Methylation-Gold Kit (Zymo Research, USA) according to the manufacturer’s instructions. Methylation analysis targeted the *Ela3B* gene (NC_000070.7: c.137,159,257–137,146,891; Mus musculus strain C57BL/6J, chromosome 4, GRCm39), *Claudin-7* (Cldn7, NC_000077.7: c.69,855,605–69,858,712; chromosome 11, GRCm39), and *Igk-ED* (Database NCBI37/mm9, chromosome 6: chr6:70,693,704–70,694,944). CpG islands were identified using the MethPrimer online tool (http://www.urogene.org/methprimer/) (Li and Dahiya 2002). Pyrosequencing primers were designed using the PyroMark Assay Design 2.0 software (Qiagen) and detailed information on primer sequences, annealing temperatures, and amplicon sizes is provided in Supplementary Table 2. PCR amplification was performed with an initial denaturation step at 95 °C for 15 min, followed by 50 cycles of 95 °C for 15 s, 60 °C for 30 s, and 70 °C for 30 s, with a final extension step at 72 °C for 10 min. Pyrosequencing was conducted using the PyroMark Q48 Autoprep instrument (Qiagen), and methylation levels at individual CpG sites were quantified using PyroMark Q48 Autoprep software version 2.0.7.

Differences in methylation levels between control and AD mice were evaluated using Student’s t-test.

### Power Analysis

Sample size for omics was determined by performing an a priori power analysis using G*Power software (version 3.1.9.7; Heinrich-Heine-Universität Düsseldorf, Germany). An independent two-tailed Student’s t-test was selected with a significance level of α = 0.05, and a desired statistical power of 0.90. Assuming a twofold modulation of the parameters of interest, the expected effect size (Cohen’s d) was estimated based on a standard deviation of 0.5 and an expected mean difference of 1, resulting in an effect size of d = 2. Under these assumptions, the required sample size was seven animals per group.

### Statistical Analysis

Statistical analyses were performed using GraphPad Prism 8.0 (GraphPad Software). Data are presented as mean ± standard deviation (SD). For miRNome profiling, statistical significance was defined using a false discovery rate (FDR, Benjamini–Hochberg) q-value < 0.05, with additional consideration of nominal p-values < 0.05 (Mann–Whitney).

Differential protein expression analysis was performed using Progenesis QI for Proteomics, in which ANOVA represents the core statistical approach. The identification algorithm was filtered using a FDR of 1%. Proteins were considered differentially expressed when both *p* ≤ 0.05 (≥ 1.3 in −log10 scale) and |log2 fold change ≥ 0.4 (corresponding to a ≥ 1.5-fold change) were satisfied.

miRCURY LNA miRNA Custom Panels were analyzed using the GeneGlobe miRNA PCR Array Data Analysis tool (Qiagen). This platform provided quality control for reverse transcription and PCR performance. Fold change values were calculated as the ratio of normalized miRNA expression in test samples relative to controls using the 2^(-ΔCt) method.

Prior to statistical testing, data distribution and variance homogeneity were evaluated using the Shapiro–Wilk test, and F test, respectively. Based on these assumptions, either an unpaired Student’s t-test or Welch’s t-test was applied. For comparisons across multiple disease stages, one-way ANOVA was used when assumptions of normality and homogeneity of variances were satisfied, followed by Tukey’s multiple comparisons post hoc test.

The specific statistical test applied in each case is indicated in the corresponding figure legends. Differences were considered statistically significant at *p* ≤ 0.05 (*), *p* ≤ 0.01 (**), *p* ≤ 0.001 (***), and *p* ≤ 0.0001 (****).

## Results

### Identification and Validation of Fecal miRNA Biomarkers Associated with AD

To characterize AD–associated alterations in fecal miRNAs, we performed small RNA sequencing on stool samples from 15-month-old 3×Tg-AD mice and age-matched controls. Sequencing detected 400 miRNAs, and principal component analysis (PCA) demonstrated a clear separation between groups (Supplementary Fig. 1). miRNome analysis, comparing expression profiles of AD vs controls, identified 31 significantly dysregulated miRNAs (*p* < 0.05), comprising 9 upregulated and 22 downregulated (Tables 1 and 2), with fold-changes ranging from + 9.27 to − 63. Only a subset of the microRNAs (*n* = 12) met the FDR significance threshold (q ≤ 0.05). However, given the relatively low complexity of the fecal miRNome dataset, we adopted a combined statistical and biological selection strategy for the following analysis.

**Table 1:** List of miRNAs upregulated in AD compared to controls

Name	Fold change	*p*-value	q-value^§^	Bonferroni
**miR-142a-5p**	**9.27**	1.50E-05	9.56E-04	0.005
**miR-223-3p**	**3.20**	1.97E-03	3.56E-02	0.679
**miR-215-3p**	**3.03**	4.46E-02	2.91E-01	1.000
**miR-183-5p**	**2.40**	2.39E-02	2.09E-01	1.000
**miR-194-1-3p**	**2.25**	1.45E-02	1.47E-01	1.000
**miR-5112**	**2.15**	5.05E-03	6.50E-02	1.000
**miR-322-5p**	**2.09**	3.82E-02	2.73E-01	1.000
miR-200a-5p	1.42	2.91E-02	2.34E-01	1.000
miR-429-3p	1.35	2.30E-02	2.09E-01	1.000

**miRNAs **shown in **bold** indicate a fold increase > 2

^§^FDR-adjusted p-values

**Table 2:** List of miRNAs downregulated in AD compared to controls

Name	Fold change	*p*-value	q-value^§^	Bonferroni
**miR-6966-5p**	**−63.00**	1.22E-02	1.31E-01	1.00
**miR-100-5p**	**−40.46**	4.19E-02	2.89E-01	1.00
**miR-335-5p**	**−18.47**	4.50E-03	6.21E-02	1.00
**miR-449a-5p**	**−12.11**	4.83E-02	3.01E-01	1.00
**miR-3968**	**−6.42**	4.52E-02	2.91E-01	1.00
**miR-20b-5p**	**−5.43**	2.48E-04	7.97E-03	0.09
**miR-8117**	**−5.38**	5.20E-05	2.07E-03	0.02
**miR-146b-5p**	**−4.46**	2.51E-02	2.11E-01	1.00
**miR-3470b**	**−4.20**	1.29E-03	2.76E-02	0.44
**miR-2137**	**−3.18**	5.12E-09	9.89E-07	1.76E-06
**miR-206-3p**	**−2.92**	3.11E-02	2.40E-01	1.00
**miR-6238**	**−2.76**	5.36E-05	2.07E-03	0.02
**miR-8109**	**−2.70**	8.76E-03	9.94E-02	1.00
**miR-6239**	**−2.60**	6.64E-04	1.83E-02	0.23
**miR-709**	**−2.53**	2.40E-03	3.86E-02	0.82
**miR-6236**	**−2.45**	4.63E-08	4.47E-06	1.59E-05
**miR-5121**	**−2.35**	7.43E-03	8.97E-02	1.00
**miR-3473a**	**−2.33**	7.82E-04	1.89E-02	0.27
miR-5126	−1.89	2.01E-02	1.94E-01	1.00
miR-1a-3p	−1.80	3.40E-02	2.53E-01	1.00
miR-6240	−1.69	3.70E-03	5.49E-02	1.00
miR-6538	−1.69	2.03E-03	3.05E-02	0.70

**miRNAs** shown in **bold** indicate a fold decrease > 2

^§^FDR-adjusted p-values

**Fig. 2:**
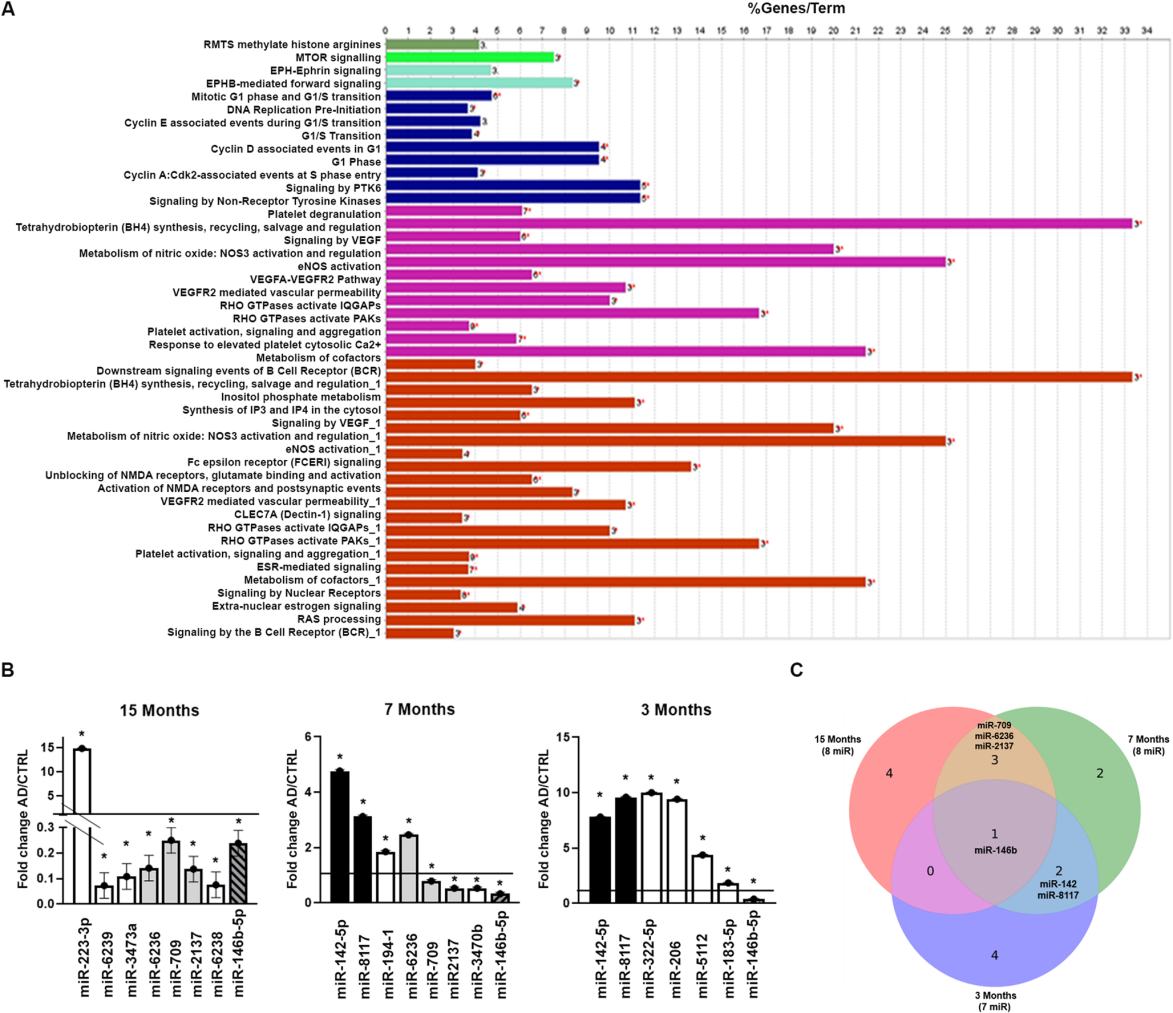
Identification and validation of molecular biomarkers for AD through fecal miRNome analysis. **A** Pathway analysis performed using Cytoscape software of the 31 miRNAs significantly differentially expressed between AD (*n* = 7) and CTRL (*n* = 6) mice identified by the miRNome analysis. **B**. Custom qPCR plates were used to validate miRNAs showing a fold change > 2 across presymptomatic (3 months), symptomatic (7 months), and severe (15 months) stages of disease. Data are presented as fold change in AD relative to CTRL. Statistical analysis was performed using unpaired, two-tailed Student’s t-tests assuming equal variances. At the severe stage (15 months), significant differences in miRNA expression were observed as follows: miR-223-3p (*p* = 0.00164), miR-6239 (*p* = 0.03586), miR-3473a (*p* = 0.00069), miR-6236 (*p* = 0.03907), miR-709 (*p* = 0.04998), miR-2137 (*p* = 0.00877), miR-6238 (*p* = 0.00590), and miR-146b-5p (*p* = 0.00355). At the symptomatic stage (7 months), the following miRNAs were significantly altered: miR-142-5p (*p* = 0.04415), miR-8117 (*p* = 0.02253), miR-194-1-3p (*p* = 0.04038), miR-6236 (*p* = 0.00869), miR-709 (*p* = 0.02736), miR-2137 (*p* = 0.02249), miR-3470b (*p* = 0.00800), and miR-146b-5p (*p* = 0.00956). At the presymptomatic stage (3 months), significant miRNAs included: miR-142-5p (*p* = 0.05020), miR-8117 (*p* = 0.02779), miR-322-5p (*p* = 0.01443), miR-206 (*p* = 0.00243), miR-5112 (*p* = 0.03553), miR-183-5p (*p* = 0.02688), and miR-146b-5p (*p* = 0.02688). Significance threshold: *p* < 0.05 (*). **C** Venn diagram showing the overlap of deregulated miRNAs identified at presymptomatic (3 months), symptomatic (7 months), and severe (15 months) disease stages

To explore potential functional relevance, predicted target genes of these miRNAs were subjected to network and pathway analysis using Cytoscape (v3.9.1) and Reactome 2021. Target genes clustered into six main signaling modules (shown by differently coloured columns in Fig. 2A), including pathways related to nitric oxide metabolism, N-methyl-D-aspartate (NMDA) receptor activity, epinephrine synthesis, and vascular endothelial growth factor (VEGF) signaling processes previously implicated in AD pathophysiology (Austin et al. 2013; Katusic and Austin 2016; Dubey et al. 2020; Liu et al. 2019; Dines and Lamprecht 2016).

From the set of 31 dysregulated miRNAs, those with a fold-change greater than 2 were selected for validation in a Custom qPCR panel (25 miRNAs, 5 housekeeping genes, and 2 positive controls). It confirmed significant modulation of eight miRNAs in 15-month-old mice: miR-146b-5p, miR-6238, miR-2137, miR-709, miR-6236, miR-3473a, miR-6239 and miR-223-3p (*p* < 0.05). All validated miRNAs except miR-223-3p were downregulated in AD samples. Several additional candidates could not be reliably quantified due to low abundance, consistent with the higher sensitivity of sequencing.

To map temporal dynamics, the Custom qPCR panel previously described was further assessed in independent cohorts representing presymptomatic (3-month), and symptomatic (7-month) disease stages. In 7-month-old mice, eight miRNAs were significantly altered, with four upregulated (miR-142-5p, miR-8117, miR-194-1, miR-6236) and four downregulated (miR-709, miR-2137, miR-3470b, miR-146b-5p). In 3-month-old presymptomatic mice, seven miRNAs exhibited significant changes: six were upregulated (miR-142-5p, miR-8117, miR-322-5p, miR-206, miR-5112, miR-183-5p), while miR-146b-5p was downregulated (*p* < 0.05; Fig. 2B). Comparative cross-stage analysis illustrated in the Venn diagram showed that most miRNAs were stage-specific (*n* = 10), five were shared between two time points (miR-142-5p, miR-8117, miR-6236, miR-709, miR-2137), and exhibited a progressive time-dependent downregulation except for miR-6236 which shows a reversed expression trajectory while miR-146b-5p was consistently dysregulated across all disease stages (Fig. 2C).

Together, these findings reveal substantial temporal remodeling of the fecal miRNA profile during AD progression and identify miR-146b-5p as a robust, stage-spanning molecular signature in stool.

### Fecal Proteomic Profiling Identifies AD-Associated Molecular Alterations

**Fig. 3:**
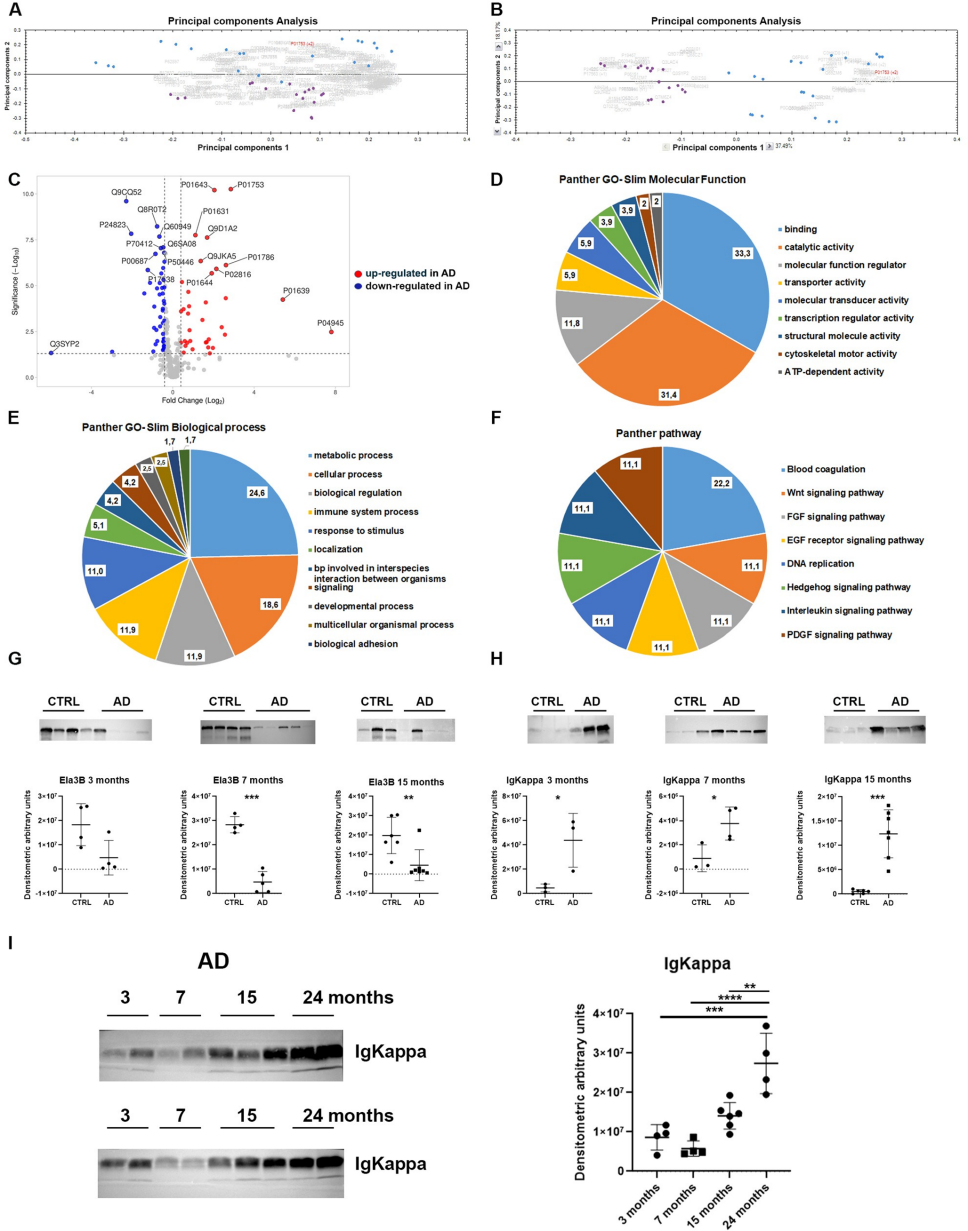
Identification and validation of molecular biomarkers for AD through fecal proteome analysis. **A** PCA of all 300 identified proteins and **B** of the subset of 81 differentially modulated proteins, comparing CTRL and AD mice (purple dots = CTRL; blue dots = AD). **C** Volcano plot of all quantified proteins. **D**–**F** Differentially modulated proteins (*n* = 81) were subjected to functional enrichment analysis using the Panther Classification System (v17.0), focusing on **D** molecular functions, **E** biological processes, and **F** signaling pathways. Validation of **G** Fecal Ela3B and **H** fecal IgKappa levels assessed by immunoblotting in CTRL (*n* = 3/4) and AD (*n* = 3/7) mice across disease stages. Immunoblot analyses were conducted after verifying data distribution (Shapiro–Wilk test) and variance homogeneity (F test). At 3 months, Ela3B expression did not differ significantly between groups (unpaired t test: t = 2.427, df = 6, *p* = 0.0514). Data were normally distributed (*p* = 0.2182), and variances were homogeneous (F = 1.474, DFn = 3, DFd = 3; *p* = 0.7576). A significant difference emerged at 7 months (unpaired t test: t = 8,921, df = 7, p = < 0,0001), with normally distributed data (*p* = 0,2696) and homogeneous variances (F = 1,643, DFn = 4, DFd = 3; *p* = 0.8465). At 15 months, Ela3B levels were significantly reduced in AD mice compared with CTRL (unpaired t test: t = 3.200, df = 11, *p* = 0.0085), with no significant difference in variances (F = 1.369, DFn = 5, DFd = 6; *p* = 0.705). Although Shapiro–Wilk indicated partial deviation from normality (*p* = 0.017), the large effect size supports the robustness of this result. For IgKappa, no significant difference was observed at 3 months (Welch’s t test: t = 3.045, df = 2.085, *p* = 0.0884). Data were normally distributed (*p* = 0.4329), but variances were unequal (F = 47.26, DFn = 2, DFd = 2; *p* = 0.0414). A significant difference emerged at 7 months (unpaired t test: t = 2.957, df = 5, *p* = 0.0316), with normally distributed data (*p* = 0.059) and homogeneous variances (F = 1.504, DFn = 3, DFd = 2; *p* = 0.8465). At 15 months fecal IgKappa levels in AD was significantly increased (unpaired t test with Welch’s correction t = 6,341, df = 6,085, *p* = 0.0007). Data were normally distributed (*p* = 0,1438), but variances were unequal (F = 165,4 DFn = 6, DFd = 5; *p* = 0.0414). **I** Fecal IgKappa levels in AD mice across disease progression (3–24 months) were further analyzed by one-way ANOVA. Significant differences were observed, by Post hoc analysis using Tukey’s multiple comparisons test, between 3 and 24 months (*p* = 0.0002), 7 and 24 months (*p* < 0.0001), and 15 and 24 months (*p* = 0.0019). The comparison between 7 and 15 months approached significance (*p* = 0.0508). Data met assumptions of normality (Shapiro–Wilk *p* = 0.9224) and homogeneity of variances (Bartlett’s statistic = 5.665, *p* = 0.129). Significance levels: * *p* < 0.05, ** *p* < 0.01, *** *p* < 0.001

To extend our analysis of stool-derived molecular signatures, we performed quantitative proteomic profiling of fecal samples from 15-month-old 3×Tg-AD and age-matched control mice. Across all samples, 300 proteins were detected, of which 81 were significantly modulated (ANOVA *p* < 0.05; fold-change > 1.5). PCA confirmed strong within-group reproducibility when considering the full proteome (Fig. 3A), whereas the subset of differentially modulated proteins yielded clear segregation between AD and control groups (Fig. 3B).

Volcano plot analysis further demonstrated 34 upregulated and 47 downregulated proteins in AD mice (Fig. 3C; Supplementary Tables 3–4).

Functional classification of 81 significantly modulated proteins using PANTHER (v17.0) revealed that binding and catalytic activities represented the most enriched molecular functions (33.3% and 31.4%, respectively; Fig. 3D). Metabolic and cellular processes were the most prominent biological categories (24.6% and 18.6%, respectively; Fig. 3E). Pathway enrichment analysis identified dysregulation in blood coagulation, Wnt, fibroblast growth factor (FGF) signaling, epidermal growth factor (EGF) receptor, DNA replication, Hedgehog, interleukin, and platelet-derived growth factor (PDGF) signaling pathways, with blood coagulation emerging as the most represented pathway (22.2%; Fig. 3F). These functional patterns are consistent with inflammatory and metabolic alterations implicated in AD pathogenesis.

To validate the proteomic findings, two of the most strongly modulated proteins, Ig kappa chain (IgKappa; upregulated) and elastase 3B (Ela3B; downregulated), were assessed by Western blotting. Both proteins were confirmed to be significantly altered in 15-month-old mice. To investigate stage-dependent dynamics, expression levels were subsequently evaluated in presymptomatic (3-month-old), early symptomatic (7-month-old), and severe (24-month-old) AD cohorts. Ela3B shows a trend in the preclinical stage, followed by a significant and consistent decrease across the subsequent disease stages. (Fig. 3G), whereas IgKappa showed a progressive increase, with early perturbations evident at 3 months and maximal levels observed at 24 months (Fig. 3H, I). These trends reinforce the involvement of digestive and inflammatory processes, previously identified as contributors to AD risk and progression (Bornemann et al. 2025; Pappolla et al. 2024), and highlight fecal proteins as potential biomarkers reflecting disease prognosis.

### Integrated miRNome–Proteome Analysis

**Fig. 4:**
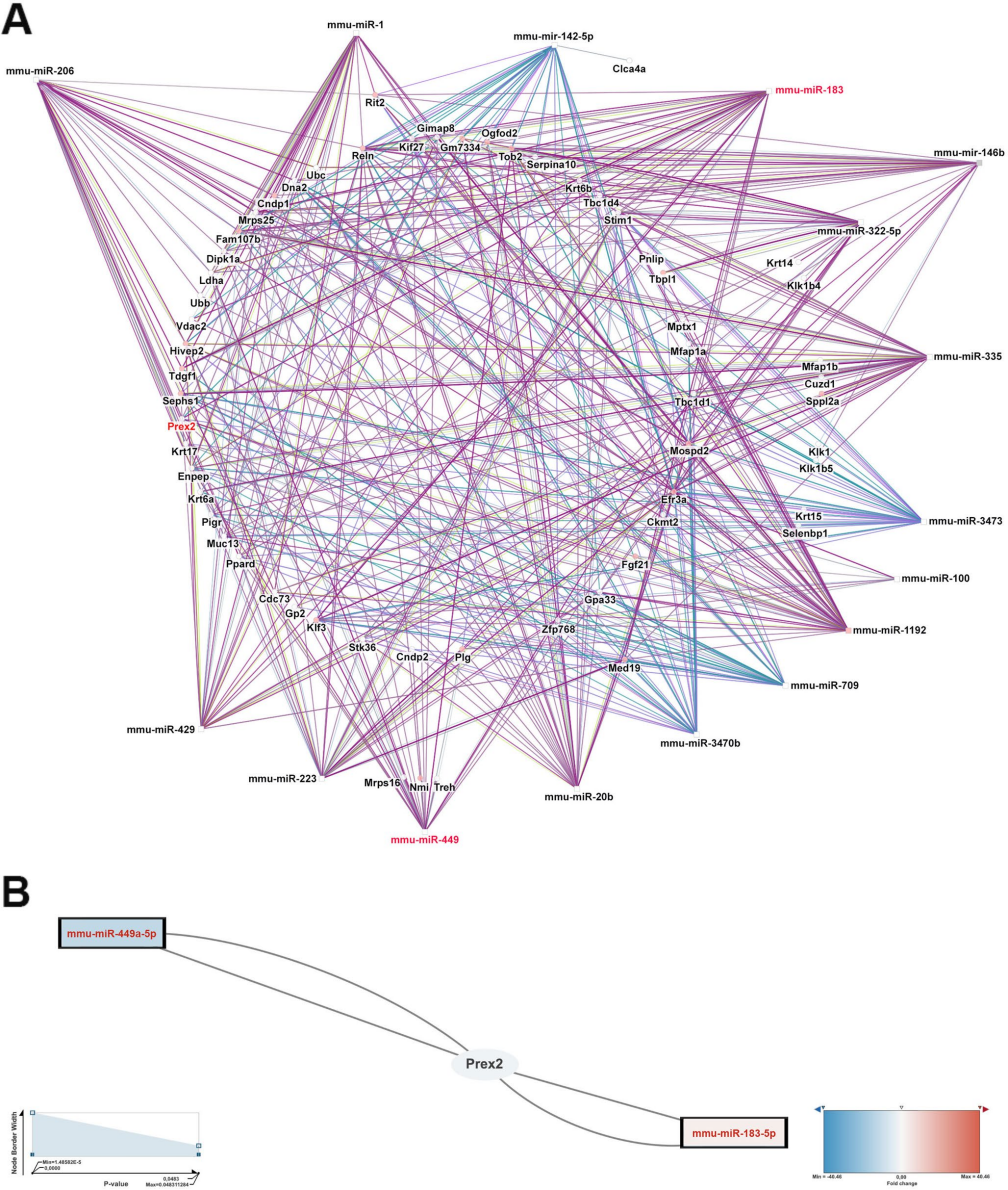
Integrated miRNome–proteome analysis. **A** miRNA–mRNA interaction network illustrating predicted regulatory relationships. **B** Interaction map depicting the relationship between the Prex2 protein and two differentially modulated miRNAs identified in this study miR-449a-5p and miR-183-5p represented as edges within the network

To further evaluate the utility of stool-derived molecules as biomarkers and to identify coordinated regulatory mechanisms, we performed an integrated analysis combining fecal miRNA and proteomic datasets. Using experimentally validated interactions from miRTarBase v9.0, we constructed a miRNA–mRNA interaction network incorporating all differentially expressed miRNAs detected in fecal samples. The resulting network (Fig. 4A) comprised 16 modulated miRNAs (miR-709, miR-449, miR-429, miR-3473, miR-3470b, miR-335, miR-322-5p, miR-223, miR-20b, miR-206, miR-183, miR-146b, miR-142-5p, miR-1192, miR-100, and miR-1) linked to 58 validated mRNA targets, including genes involved in metabolic regulation, epithelial function, and immune responses (e.g., Vdac2, Ubc/Ubb, Tbc1d1/Tbc1d4, Stim1, Serpina10, Reln, Ppard, Plg, Muc13, Med19, and Enpep).

Among all predicted targets, only Prex2 (phosphatidylinositol-3,4,5-trisphosphate–dependent Rac exchange factor 2) encoded a protein that was significantly altered at the proteomic level in 3×Tg-AD mice. As shown in Tables 1, 2 and 4, Prex2 was significantly downregulated (fold change = − 1.62; *p* = 0.032) and was targeted by two miRNAs that were also modulated in our dataset: miR-449a-5p (downregulated; fold change = − 12.11; *p* = 0.048) and miR-183-5p (upregulated; fold change = 2.40; *p* = 0.024). These regulatory relationships are depicted in the integrated miRNA–protein subnetwork (Fig. 4B).

The biological relevance of this finding is supported by previous evidence that identified rare pathogenic Prex2 variants as AD risk (Xiong et al. 2022).

### Morphological, Molecular and Epigenetic Characterization of the Intestinal Epithelium

**Fig. 5:**
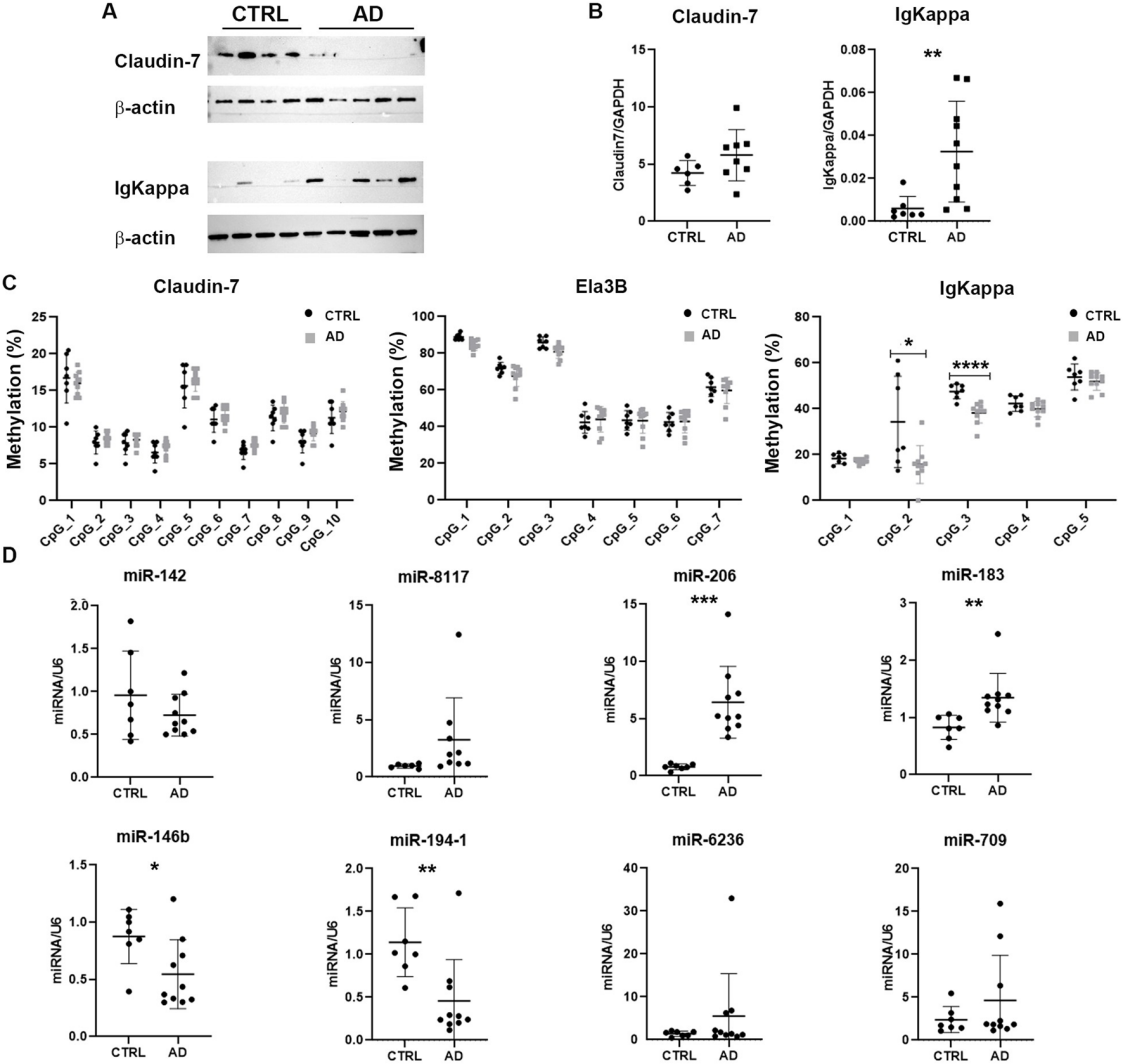
Molecular characterization of the intestinal epithelium. **A** Representatives immunoblot analysis of Claudin-7 and IgKappa protein levels and **B** their gene expression analysis. Before statistical analysis, data distribution was assessed using the Shapiro–Wilk test and variance homogeneity using the F test. According to these assumptions, either a standard unpaired t test or an unpaired t test with Welch’s correction was applied. Claudin-7 expression did not differ significantly between groups (unpaired t test: t = 1.567, df = 12, *p* = 0.1430). Data were normally distributed (Shapiro–Wilk *p* = 0.6256) and variances were homogeneous (F = 4.300, DFn = 7, DFd = 5; *p* = 0.126). In contrast, IgKappa expression was significantly different between groups (Welch-corrected t test: t = 3.426, df = 10.43, *p* = 0.0061). Data were normally distributed (Shapiro–Wilk *p* = 0.3046), but variances were unequal (F = 17.45, DFn = 9, DFd = 6; *p* = 0.0024). **C** Epigenetic profiling of Claudin-7, Ela3B, and IgKappa.IgKappa methylation at CpG_2 and CpG_3 showed significant differences between groups. For CpG_2, Welch-corrected t test showed significance (t = 2.664, df = 15, *p* = 0.0177); data were normally distributed (Shapiro–Wilk *p* = 0.3897) with unequal variances (F = 5.817, DFn = 6, DFd = 9; *p* = 0.0198). For CpG_3, a standard unpaired t test revealed a highly significant difference (t = 4.801, df = 15, *p* = 0.0002); data were normally distributed (Shapiro–Wilk *p* = 0.0551) and variances were homogeneous (F = 1.875, DFn = 9, DFd = 6; *p* = 0.4575). **D** miRNA analysis was also preceded by assessment of normality and variance homogeneity. Depending on these results, either a standard unpaired t test or Welch’s correction was applied. miR-142: not significant (Welch: t = 1.119, df = 7.878, *p* = 0.2962); normal distribution (*p* = 0.2835), unequal variances (F = 4.517; *p* = 0.0438). miR-8117: not significant (Welch: t = 1.858, df = 8.059, *p* = 0.1000); non-normal distribution (*p* < 0.0001), unequal variances (F = 409.5; *p* < 0.0001). miR-206: significant (Welch: t = 5.654, df = 9.168, *p* = 0.0003); non-normal distribution (*p* = 0.0011), unequal variances (F = 153.0; *p* < 0.0001). miR-183: significant (standard t test: t = 2.969, df = 15, *p* = 0.0096); non-normal distribution (*p* = 0.0024), homogeneous variances (F = 4.024; *p* = 0.1042). miR-146b: significant (standard t test: t = 2.417, df = 15, *p* = 0.0289); normal distribution (*p* = 0.6827), homogeneous variances (F = 1.607; *p* = 0.5803). miR-194-1: significant (standard t test: t = 3.086, df = 15, *p* = 0.0075); non-normal distribution (*p* = 0.0040), homogeneous variances (*p* = 0.6851). miR-6236: not significant (Welch: *p* = 0.2214); non-normal distribution (*p* < 0.0001), unequal variances (F = 271.4; *p* < 0.0001). miR-709: not significant (Welch: t = 1.272, df = 11.05, *p* = 0.2294); non-normal distribution (*p* = 0.0012), unequal variances (F = 11.91; *p* = 0.0069). All analyses were performed in colon of 24-old CTRL (*n* = 7) and AD (*n* = 10) mice, Significance levels: *p* < 0.05 (*), *p* < 0.01 (**), *p* < 0.001 (***), *p* < 0.0001 (****)

To assess whether fecal biomarker alterations correspond to changes in gut physiology, we performed comprehensive histological, molecular, and epigenetic analyses of colon tissue from 24-month-old 3×Tg-AD and age-matched control mice. Histological evaluation revealed no significant differences in macrophage infiltration (IBA1 staining) or goblet cell distribution (Supplementary Fig. 2A–B). Similarly, levels of tight junction ZO-1 were unchanged (data not shown). In contrast, immunoblotting demonstrated a significant reduction of Claudin-7 expression in AD mice (Fig. 5A), suggesting slight impairment of epithelial barrier integrity.

Consistent with fecal proteomic data, IgKappa expression was markedly elevated at both transcript and protein levels in AD colon tissues (Fig. 5A, B), whereas Ela3B remained undetectable. Notably, Claudin-7 transcript abundance did not differ between groups, indicating post-transcriptional regulation. DNA methylation analysis revealed no changes at the *Claudin-7* or *Ela3B* loci but showed significant hypomethylation of CpG2 and CpG3 sites (*p* < 0.01 and *p* < 0.001, respectively) within the IgKappa enhancer region (Fig. 5C), suggesting that an epigenetic mechanism may underly its upregulation in AD mice and explain its increase in AD fecal samples. These findings indicate that intestinal barrier dysfunction and local immune modulation may contribute to AD pathophysiology.

To investigate whether fecal miRNA dysregulation reflects changes in the intestinal epithelium, we examined eight miRNAs with human homologues in colon tissue. Four miRNAs were significantly altered in AD mice. Three—miR-146b, miR-206, and miR-183—showed expression patterns concordant with fecal samples, whereas miR-194-1 exhibited an inverse trend (Figs. 2B and 5D). Collectively, these results demonstrate that a subset of fecal miRNA alterations mirror molecular changes within the intestinal epithelium.

## Discussion

AD is a leading cause of neurodegenerative dementia and disability in the aging population (Zhang et al. 2025). With projections estimating more than 100 million people affected by 2050 (Estimation of the global prevalence of dementia in 2019 and forecasted prevalence in 2050: an analysis for the Global Burden of Disease Study 2019–2022), and the absence of therapies capable of halting or reversing disease progression (Cummings et al. 2023), the development of reliable biomarkers remains a critical clinical and research priority. Indeed, current AD diagnostic methods such as neuroimaging, and CSF biomarkers are often expensive and invasive. Furthermore, patients typically undergo these diagnostic tests only when the disease has progressed to an advanced stage. Current biomarker research largely focuses on serum and CSF. Recent studies highlight the advantages of integrating CSF and serum proteomic data: Liu et al. (Liu et al. 2023) identified three CSF proteins (AT1B1, SRGN, PRDX3) with strong diagnostic performance, showing improved accuracy when combined with serum profiles. Additional studies (Roveta et al. 2024; Tao et al. 2024; Salvadò et al. 2024) further support the usefulness of CSF and serum proteins for early detection and disease monitoring. miRNAs are also gaining prominence as AD biomarkers, with strong diagnostic accuracy in blood-based assays (Zhang et al. 2019) and a mechanistic role in disease pathogenesis (Wang et al. 2019). Blood-based biomarkers are emerging as a promising and minimally invasive tool for the diagnosis of AD. Among these, phosphorylated tau isoforms (p-tau217 and p-tau181) (Palmqvist et al. 2020; Janelidze et al. 2020) and the plasma amyloid-beta ratio (Aβ42/Aβ40) (Schindler et al. 2019), have demonstrated high diagnostic accuracy and strong correlation with tau pathology and cerebral amyloid deposition. In 2025, the US Food and Drug Administration (FDA) approved the first blood-based biomarker tests intended to aid the assessment of AD (Healey 2026). Further validation and standardization across laboratories and populations before routine use in general clinical practice.

In this context, the fecal markers identified in this study may represent not invasive test able to predict early-stage disease offers a novel, accessible approach that could complement existing diagnostics, enabling earlier detection and intervention and helping to avoid invasive analyses. The integration of serum- and fecal-based biomarkers may represent a novel and promising strategy to enhance diagnostic performance, as well as to enable more accurate monitoring of therapeutic responses.

Recent studies have leveraged fecal samples for integrated metagenomic and metabolomic analyses, uncovering consistent microbiota and metabolome alterations in AD. For example, Zhao et al. applied 16 S rDNA sequencing alongside untargeted fecal metabolomics in AD, mild cognitive impairment, and control groups, revealing progressive shifts in microbial taxa (e.g., elevated *Staphylococcus* and *Bacillus*, reduced *Anaerostipes*) and metabolites (e.g., arachidonic, adrenic, lithocholic acids), which correlated with neuroimaging markers and cognitive decline (Zhao et al. 2025). Similarly, animal-model studies (APP/PS1 mice) revealed gut dysbiosis and fecal metabolome shifts, including lipid perturbations, that mirrored serum and brain metabolite profiles, confirming a strong gut–metabolite–brain axis in AD (Xin et al. 2019). These findings highlight fecal multi-omics as a non-invasive window into functional microbiota–metabolite alterations in AD, offering mechanistic insights into gut–brain interactions in dementia.

However, while previous studies investigating the gut–brain axis in AD have primarily focused on the gut microbiome and metabolome, these approaches often rely on complex analytical pipelines and generate highly variable datasets that can be influenced by several external factors, including diet, medication, and environmental conditions. Moreover, the translation of microbiome- or metabolome-based signatures into standardized diagnostic assays remains challenging. In contrast, miRNAs and proteins represent molecular entities that can be reliably quantified using well-established and widely available techniques commonly used in clinical practice, such as quantitative PCR and immunoassays. These methods provide robust and reproducible quantification and are more easily adaptable to routine diagnostic workflows.

However, fecal samples have not yet been exploited for host-derived miRNome and proteomic biomarker discovery.

In this study, we applied multi-omic analysis of fecal samples from 3×Tg-AD mice to explore molecular alterations and identify potential biomarkers. Our data revealed 31 deregulated miRNAs and 81 proteins associated with AD, some of which displayed stage-specific patterns. Pathway enrichment of dysregulated miRNAs confirmed involvement of oxidative stress, synaptic dysfunction, neurodegeneration, and VEGF signaling, aligning with prior evidence in AD pathology. Nitric oxide metabolism regulates neuronal communication and neuroprotection, and its dysregulation contributes to oxidative stress, a critical factor in AD (Azargoonjahromi 2023). Similarly, NMDA receptor–mediated signaling is essential for synaptic plasticity, with dysregulation leading to excitotoxicity and cognitive deficits (Wang and Reddy 2017). Epinephrine pathways modulate neuroinflammation and amyloid-β/tau interactions (Gutiérrez et al. 2022; Zhang et al. 2020), while VEGF signaling maintains cerebral vasculature and neuroprotection (Ali and Bracko 2022). These findings are consistent with previously reported pathway analyses (Austin et al. 2013; Katusic and Austin 2016; Dubey et al. 2020; Liu et al. 2019; Dines and Lamprecht 2016).

Focusing on disease progression, AD mice at presymptomatic stage exhibited upregulation of miR-142-5p, miR-8117, miR-322-5p, miR-206, miR-5112, and miR-183-5p, whereas miR-146b-5p was downregulated. Notably, several of these miRNAs are altered in blood from AD patients (Xie et al. 2015; Sørensen et al. 2016; Wei et al. 2020). miR-142-5p is abnormally expressed in AD brain, and its inhibition improves cognition via Protein Tyrosine Phosphatase Non-Receptor Type 1 (PTPN1)-mediated Akt signaling (Liang et al. 2023a, 2023b). miR-206 predicts mild cognitive impairment-to-dementia progression by suppressing brain-derived neurotrophic factor expression (Kenny et al. 2019; Tian et al. 2014). miR-183-5p has been detected in CSF and serum of AD patients, with defective biogenesis linked to memory decline (Burgos et al. 2014; Jawaid et al. 2019). miR-322-5p is associated with apoptosis modulation in AD (Ma et al. 2017), while miR-5112, may regulate neuroinflammation via IKKγ inhibition (Kang et al. 2022). Interestingly miR-5112 and miR-8117 have never been associated with AD pathogenesis. miR-146b-5p mediates microglial homeostasis (Bokobza et al. 2022) and its hypermethylation pattern contributes to downregulation in the brain of AD patients (Villela et al. 2016). During the symptomatic stage, some miRNAs maintained early deregulation (miR-142-5p, miR-8117, miR-146b-5p) and miR-8117 continued to increase, while others showed stage-specific modulation, including upregulation of miR-194-1-3p and miR-6236, and downregulation of miR-3470b, miR-2137, and miR-709. This bidirectional modulation reflects progressive pathway activation or silencing. Thoroughly miR-6236, miR-709, and miR-2137 were shared between symptomatic and severe stages, with miR-6236 inverting expression in severe disease, highlighting stage-dependent functional shifts. Interestingly, miR-6236 enhances insulin signaling and prevents hyperglycemia (Paneru et al. 2024); moreover, insulin resistance is associated with AD (Matsuzaki et al. 2010). Notably, for miR-2137 and miR-3470b no evidence supporting an association with AD has been reported prior to this study, only functional association with stroke (Cai et al. 2019), while miR-709 was previously associated with cognitive decline (Yamamoto et al. 2024). At AD severe stage most miRNAs showed pronounced decrease, except for miR-223-3p, suggesting a systematic deterioration or shutdown of the cellular processes involved. Notably, miR-223-3p was uniquely upregulated in severe AD, consistent with neural-derived extracellular vesicle signatures in patients (Serpente et al. 2020; Visconte et al. 2023).

Overall, miR-146b-5p is inversely associated with AD severity, showing a progressive decrease from asymptomatic to severe stages. In addition, several miRNAs—including miR-142-5p, miR-8117, miR-709, miR-6236—were significantly altered across different disease stages, supporting their potential utility as biomarkers for early diagnosis and prognosis.

Proteomic analysis revealed enrichment in blood coagulation, Wnt, FGF, PDGF, and interleukin signaling. Wnt/β-catenin maintains blood–brain barrier (BBB) integrity, synaptic plasticity, and neuronal survival; its dysregulation contributes to Aβ accumulation and neurotoxicity in AD. Activation of Wnt signaling shows neuroprotective effects in both in vitro and in vivo models (Tapia-Rojas and Inestrosa 2018; Wang et al. 2022). Thrombin-mediated coagulation promotes neuroinflammation, with inhibitors mitigating AD pathology (Iannucci and Grammas 2023). FGF-2 and PDGF-BB support neurogenesis and synaptic maintenance (Mashayekhi et al. 2010).

Additional analyses were conducted to validate, in an independent cohort of fecal samples, two proteins, IgKappa and Ela3B, identified in the fecal stream. These proteins are involved in processes such as inflammation, digestive functions and dyslipidemia, identified as a risk factor for AD (Bornemann et al. 2025; Pappolla et al. 2024). IgKappa, a B-cell immunoglobulin component, is elevated in AD and may modulate neuroinflammation, consistent with evidence of B-cell infiltration and IgG deposition near Aβ plaques in transgenic mice. Depletion of B cells (genetically or via anti-CD20 antibodies) reduces plaque burden, ameliorates memory deficits, and shifts microglia toward a neuroprotective phenotype (Kim et al. 2021). Studies in humans show altered B‑cell profiles with elevated autoantibody-producing plasma B cells targeting Aβ−42 protofibrils in AD patients (Söllvander et al. 2015). Conversely, another research suggests that certain B cell subsets might have protective or regulatory roles, potentially influence microglial activity or maintain immune balance (Gol Mohammad Pour Afrakoti et al. 2025).

The other protein identified by proteomics and found to be decreased in feces, chymotrypsin-like elastase family member 3B (CELA3B, also known as elastase-3B or Ela3B), is a pancreatic enzyme involved in protein digestion and cholesterol metabolism (Sziegoleit 1982). Its reduction could suggest a potential link between AD and cholesterol dysregulation (Ibengwe and Suzuki 1987; Ishii 2019). In our study, we observed a consistent decrease in fecal Ela3B across all stages of AD, along with a progressive increase in IgKappa levels as the disease advanced. Combined, these proteins may serve as complementary biomarkers for early diagnosis and prognosis.

Integration of miRNome and proteome data identified *Prex2*, a gene coding for a protein downregulated in AD and targeted by miR-449a-5p and miR-183-5p, as a potential pathogenic gene (Xiong et al. 2022). Additional studies have linked Prex2 to vascular abnormalities, including arteriovenous malformations associated with cerebral amyloid-β deposition,a recognized contributor to AD pathogenesis (Wang et al. 2018; Hart et al. 1988; Tsonou 2020), supporting the translational relevance of fecal molecular profiling.

Colon tissue analysis revealed no major morphological alterations in AD mice at 24 months, contrasting with earlier reports regarding goblet cell distribution (He et al. 2023), although temporal differences likely account for this discrepancy. Molecular analyses indicated reduced Claudin-7, suggesting increased permeability, and strong IgKappa upregulation driven by enhancer hypomethylation, providing novel insights into its regulatory mechanisms in AD. Ela3B was absent from colon tissue, consistent with its pancreas-restricted expression. The reduction observed in fecal samples from AD mice likely reflects altered pancreatic secretion or tissue-specific downregulation. Consistently, previous studies, show that the 3×Tg-AD mouse model exhibits an age-dependent impairment of glucose tolerance driven by pancreatic amyloidopathy, highlighting a mechanistic link between neurodegeneration and metabolic dysfunction (Vandal et al. 2015). Of eight miRNAs tested, four were modulated in colon tissue, three of which mirrored fecal trends (miR-206, miR-183, miR-146b), supporting a mechanistic link between intestinal alterations and fecal biomarkers. The congenic footprint of the 3×Tg-AD mouse model should be acknowledged as a possible limitation of this study, which may have influenced the phenotypic outcomes (Schalkwyk et al. 2007; Kõks et al. 2009). Nonetheless, the consistent, stage-dependent molecular changes observed, validated across independent cohorts, and partially reflected in intestinal tissues support the biological relevance of these models to AD pathology. Validation of the miRNA signature identified in the present study can be easily conducted in future studies using human fecal samples from patients with AD at different stages of pathology.

## Conclusions

Collectively, our findings establish fecal multi-omics as a robust and non-invasive platform for AD biomarker discovery, capable of capturing stage-specific molecular signatures linked to neuroinflammation, synaptic dysfunction, and metabolic dysregulation. A 16-miRNA fecal signature, delineated disease progression from presymptomatic to severe stages, supporting its potential utility for early diagnosis and longitudinal monitoring. Notably, miR-146b-5p displayed the most stable and reproducible trajectory, underscoring its promise as a core component of a fecal biomarker panel.

In parallel, IgKappa and Ela3B emerged as complementary protein biomarkers with opposing and progressive modulation, collectively reflecting immune activation and metabolic dysfunction during disease evolution. Importantly, fecal molecular signatures were mirrored by epithelial, epigenetic, and inflammatory alterations in intestinal tissue, providing mechanistic support for their biological relevance and host origin.

Together, these data position fecal multi-omics as a minimally invasive, cost-effective, and scalable approach with strong translational potential for AD screening and disease monitoring.

Further validation in human cohorts will be required to assess clinical performance and enable translation of this strategy into actionable diagnostic and prognostic use in neurodegenerative disease.

## Supplementary Information

Below is the link to the electronic supplementary material.

Supplementary Material 1

Supplementary Material 2

Supplementary Material 3

Supplementary Material 4

Supplementary Material 5

Supplementary Material 6

Supplementary Material 7

## Data Availability

The RNA-seq data generated in this study have been deposited in the NCBI BioProject database under accession number PRJNA1392460. The proteomics data generated in this study have been deposited in the PRIDE repository under accession number PXD070630 (DOI: 10.6019/PXD070630).

## Declarations

### Declaration of Generative Al and Al-Assisted Technologies in the Writing Process

During the preparation of this work the authors used ChatGPT in order to improve readability. After using this tool, the authors reviewed and edited the content as needed and take full responsibility for the content of the publication.
