# Supplementary figures and images for: Fecal miRNome and Proteome Profiling Uncovers Stage-Specific Biomarkers of Alzheimer’s Disease in 3×Tg-AD Mice
Source: Cell Mol Neurobiol. 2026 May 11;46:108. doi: 10.1007/s10571-026-01735-5 (PMC13332079; doi:10.1007/s10571-026-01735-5)

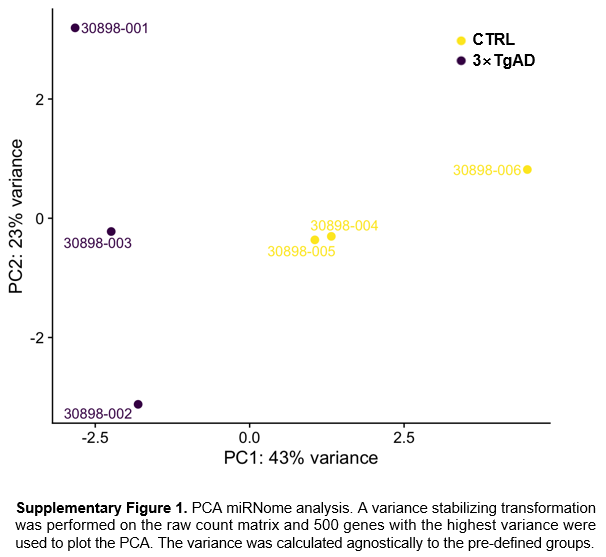

Supplement: Supplementary file 1 — Supplementary Material 1 [file 10571_2026_1735_MOESM1_ESM.tif]

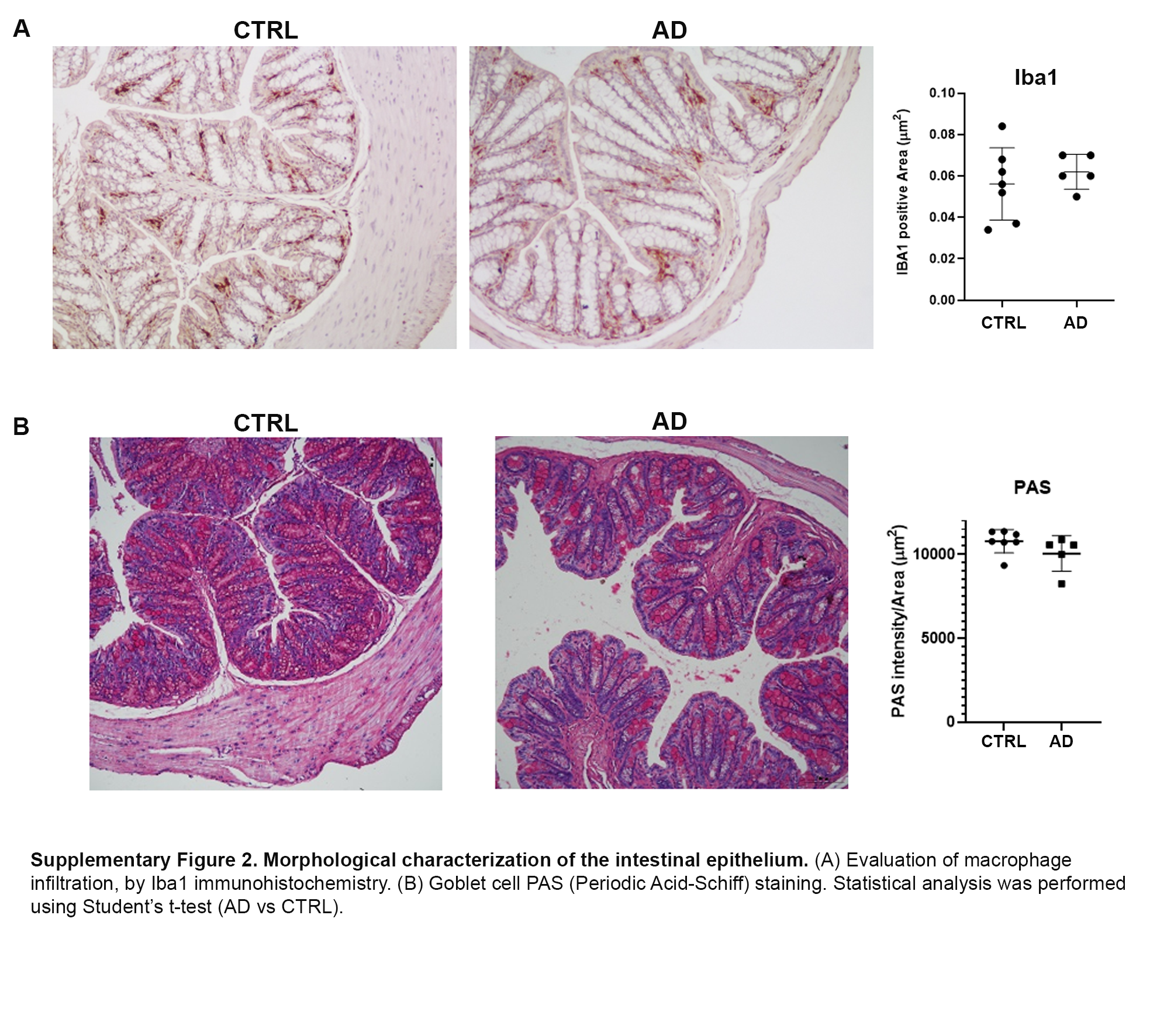

Supplement: Supplementary file 2 — Supplementary Material 2 [file 10571_2026_1735_MOESM2_ESM.tif]
