# Supplementary material for: Fecal miRNome and Proteome Profiling Uncovers Stage-Specific Biomarkers of Alzheimer’s Disease in 3×Tg-AD Mice
Source: Cell Mol Neurobiol. 2026 May 11;46:108. doi: 10.1007/s10571-026-01735-5 (PMC13332079; doi:10.1007/s10571-026-01735-5)
Supplement: Supplementary file 3 — Supplementary Material 3 [file 10571_2026_1735_MOESM3_ESM.docx]

| **Gene** | **Primer direction** | **Sequence (5’-3’)** |
| --- | --- | --- |
| *Ela3B* | Forward | GGTGGACTATGAACACTGCTCC |
| *Ela3B* | Reverse | AATCTGAGCGGGACAGTTGAGG |
| *Claudin-7* | Forward | CTGCCTTGGTAGCATGTTCCTG |
| *Claudin-7* | Reverse | CCAGCCGATAAAGATGGCAGG |
| *IgKappa* | Forward | CGGGCAAGTCAGGACATTCA |
| *IgKappa* | Reverse | CAGACTCAAGGCTGCCGATA |
| *GAPDH* | Forward | AACTTTGGCATTGTGGAAGG |
| *GAPDH* | Reverse | CACATTGGGGGTAGGAACAC |

**Supplementary Table 1**. List of primers used for Real-time PCR.
