# Supplementary material for: Fecal miRNome and Proteome Profiling Uncovers Stage-Specific Biomarkers of Alzheimer’s Disease in 3×Tg-AD Mice
Source: Cell Mol Neurobiol. 2026 May 11;46:108. doi: 10.1007/s10571-026-01735-5 (PMC13332079; doi:10.1007/s10571-026-01735-5)
Supplement: Supplementary file 4 — Supplementary Material 4 [file 10571_2026_1735_MOESM4_ESM.docx]

| **Gene** | **CpG_n_** | **Primer direction** | **Sequence (5’-3’)** | **Annealing Temperature** | **Amplicon Size [base pair (bp)]** |
| --- | --- | --- | --- | --- | --- |
| *Ela3B-F* | 7 | Forward | TGGTTGTTTTGGAGTTTATTTTGTAGA | 58°C | 239 bp |
| *Ela3B-R* |  | Reverse | BIO-AACTCAAACCTATAATCCCTATACTT |  |  |
| *Ela3B-S1* |  | Sequencer | TTTGTAGATTAGGTTGGT |  |  |
| *Claudin-7-F* | 10 | Forward | AGGTTTTGGTAGGAGATTAAAGAA | 58°C | 199 bp |
| *Claudin-7-R* |  | Reverse | BIO-ACCCTACAAACTCCAAATAACC |  |  |
| *Claudin-7-S1* |  | Sequencer | AAAAAAGAGTTGAGAGTG |  |  |
| *IGK-ED-F* | 5 | Forward | GTTATTTGAGAATATGGGGATATTTTTGAT | 58°C | 153 bp |
| *IGK-ED-R* |  | Reverse | BIO-CCCACACTCATAAAACTTACTCACTT |  |  |
| *IGK-ED-S1* |  | Sequencer_1 | ATATTTTTGATTTGAGTAGTTAT |  |  |
| *IGK-ED-S2* |  | Sequencer_2 | GGTAGTATTTAGGAGAGTAGG' |  |  |

**Supplementary Table 2**. List of genes and primers used for methylation analysis by pyrosequencing. All reverse primers are conjugate with biotin (BIO) at the 5’ end (SIAL Group, Rome, Italy). For each gene, number of CpG site analysed, annealing temperature and amplicon size are indicated.
