# Supplementary material for: Fecal miRNome and Proteome Profiling Uncovers Stage-Specific Biomarkers of Alzheimer’s Disease in 3×Tg-AD Mice
Source: Cell Mol Neurobiol. 2026 May 11;46:108. doi: 10.1007/s10571-026-01735-5 (PMC13332079; doi:10.1007/s10571-026-01735-5)
Supplement: Supplementary file 7 — Supplementary Material 7 [file 10571_2026_1735_MOESM7_ESM.pdf]

Immunoblot analyses shown in Figure 3G

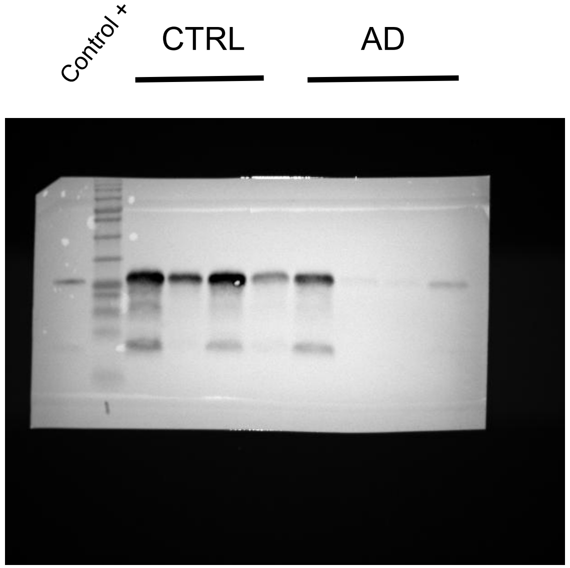

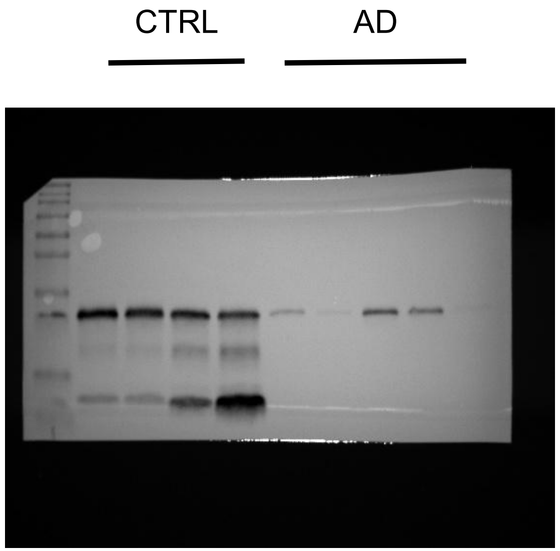

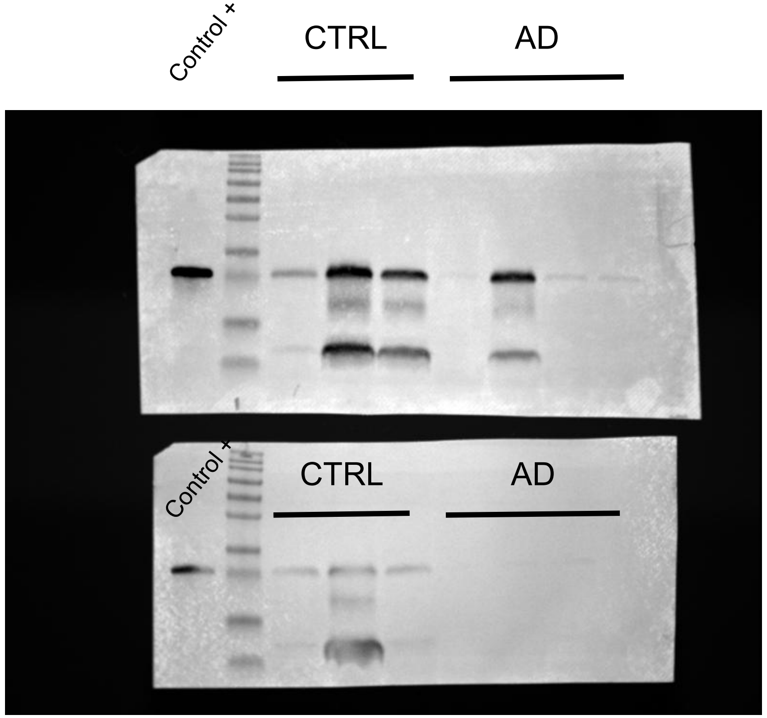

Immunoblot analyses shown in Figure 3H

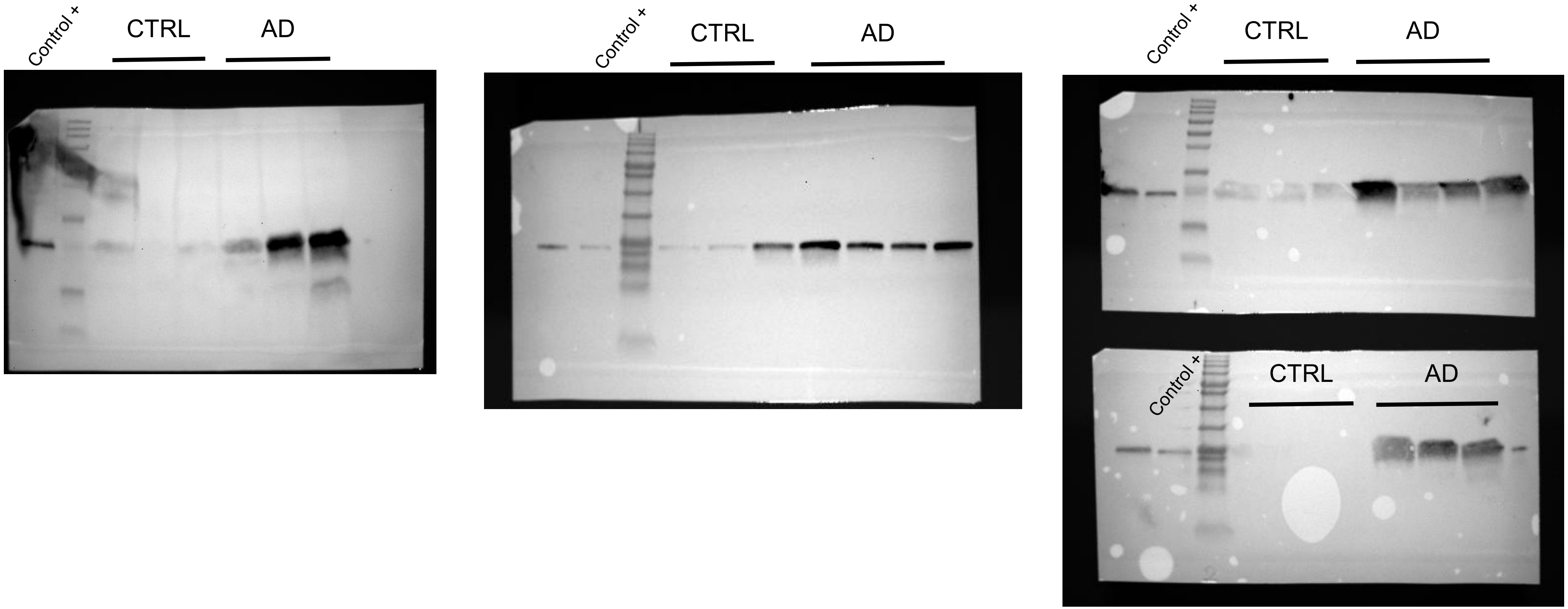

Immunoblot analyses shown in Figure 3I

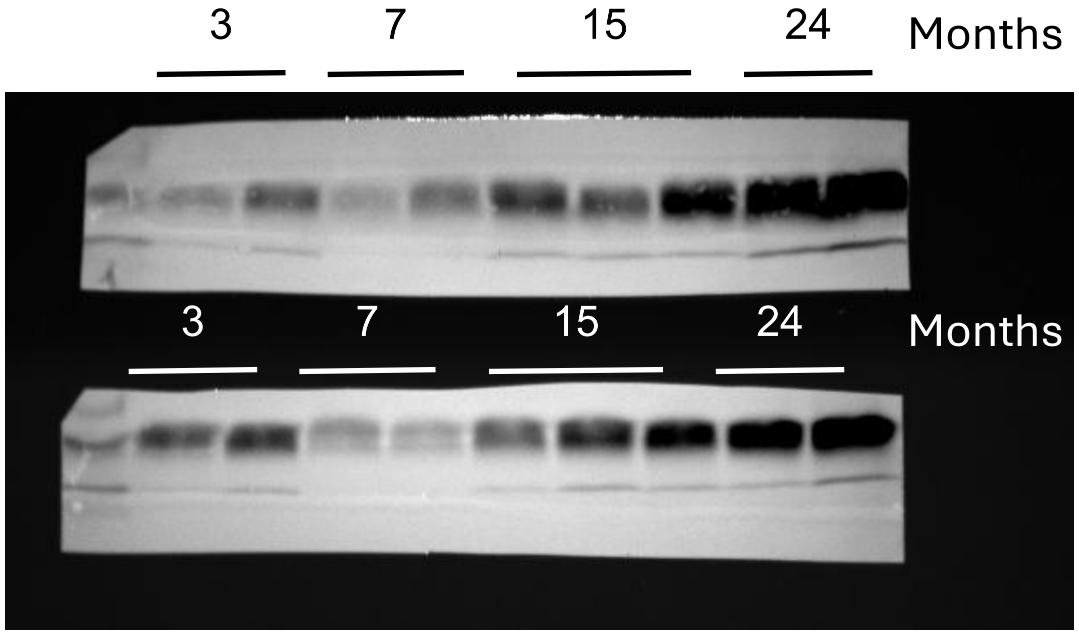

Immunoblot analyses shown in Figure 5A

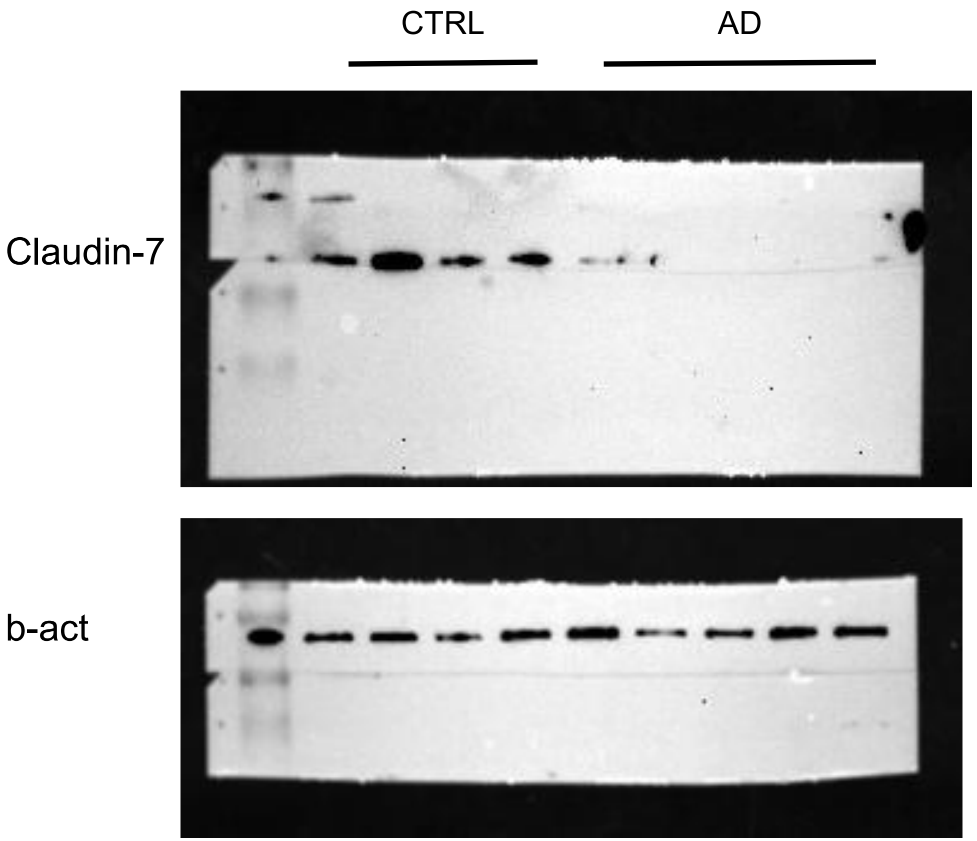

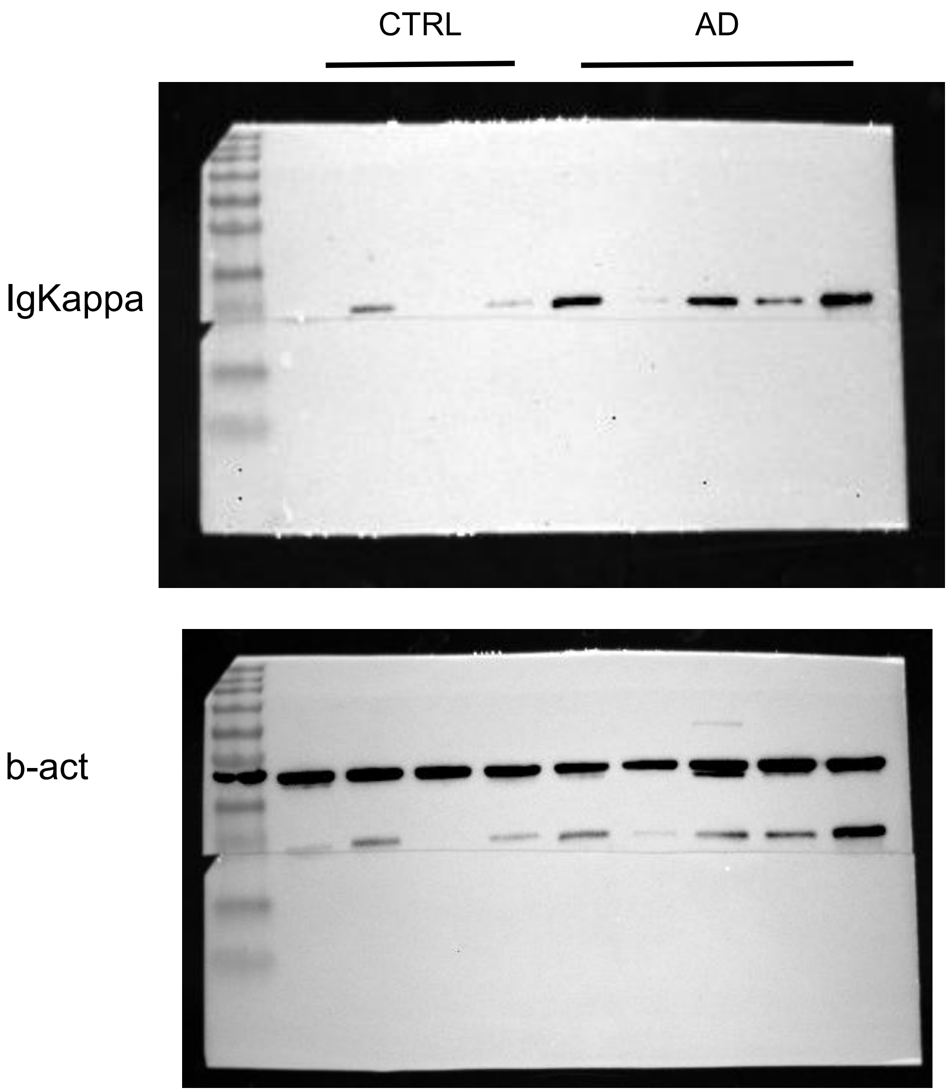

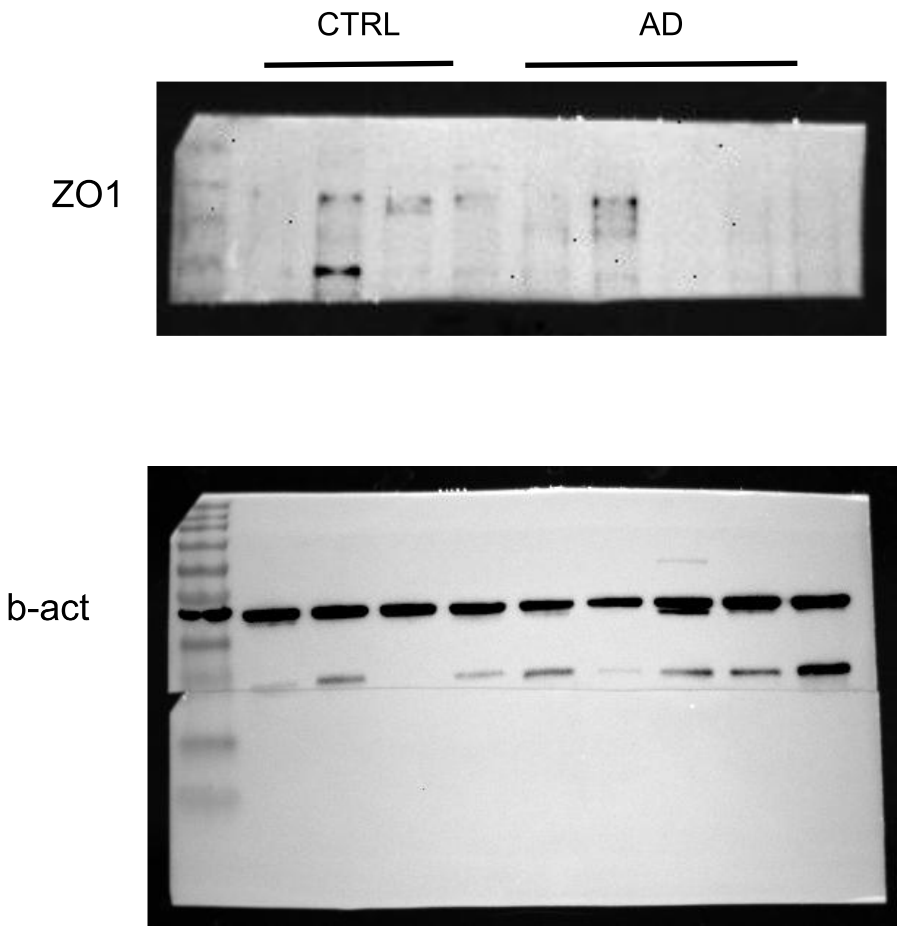

CTRL
AD
ZO1
b-act
